# Atteinte synchrone pulmonaire et des glandes lacrymales par un lymphome de type MALT

**DOI:** 10.11604/pamj.2018.29.198.14007

**Published:** 2018-04-05

**Authors:** Rim Batti, Feryel Letaief, Haifa Rachdi, Asma Zidi, Sonia Sghaier, Mouna Ayadi, Khadija Meddeb, Amina Mokrani, Yosra Yahyaoui, Henda Raies, Nesrine Chraiet, Amel Mezlini

**Affiliations:** 1 Service d’Oncologie Médicale, Institut Salah Azaiz, Tunis, Tunisie; 2 Service de Radiologie, Institut Salah Azaiez, Tunis, Tunisie

**Keywords:** Lymphome pulmonaire primitif, glande lacrymale, tissu lymphoïde associé aux muqueuses, Primary pulmonary lymphoma, lachrymal gland, lymphoid tissue associated with mucous membranes

## Abstract

Les lymphomes pulmonaires primitifs sont des tumeurs rares représentant moins de 1% des tumeurs malignes du poumon. La forme la plus fréquente est le lymphome de type Mucosa-Associated Lymphoid Tissue (MALT). L'atteinte oculaire est aussi rare et elle est dans la plupart du temps localisée dans les glandes lacrymales. Nous rapportons l'observation d'un patient ayant présenté un lymphome pulmonaire de type MALT associé à une atteinte synchrone des glandes lacrymales. Cette observation illustre les aspects cliniques, radiologiques et évolutifs de cette entité qui sont peu spécifiques.

## Abstract (en)

Primary lung lymphomas are rare tumors representing less than 1% of malignant tumors of the lung. The most frequent form is the mucosa-associated lymphoid tissue (MALT). Ocular involvement is also rare and it is mostly located in the lachrymal glands. We report the case of a patient with pulmonary MALT lymphoma associated with synchronous involvement of the lachrymal glands. This study illustrates the nonspecific clinical, radiological and evolutionary features of this disease.

## Introduction

Les lymphomes pulmonaires primitifs (LPP) sont des affections rares représentant 0,5 à 1% des tumeurs malignes primitives du poumon et 3 à 4% des lymphomes malins non hodgkiniens extra-ganglionnaires. Le lymphome pulmonaire de type MALT représente plus de la moitié des LPP de bas grade. Les manifestations cliniques et les éléments radiologiques sont peu spécifiques et le diagnostic repose essentiellement sur les analyses histologiques et immunohistochimiques des biopsies bronchiques ou pulmonaires [1, 2]. Les localisations extra-pulmonaires les plus fréquentes sont l'estomac puis les glandes lacrymales. En Tunisie, seuls quelques cas de LPP de type MALT ainsi que de rares cas de lymphome MALT de localisation lacrymale ont été décrits. Nous rapportons ainsi un cas confirmé de LPP de type MALT associé à une atteinte synchrone des glandes lacrymales pris en charge dans notre centre. A travers cette observation nous proposons d'étudier les aspects cliniques, radiologiques et évolutifs de cette entité peu spécifique.

## Patient et observation

**Figure 1:**
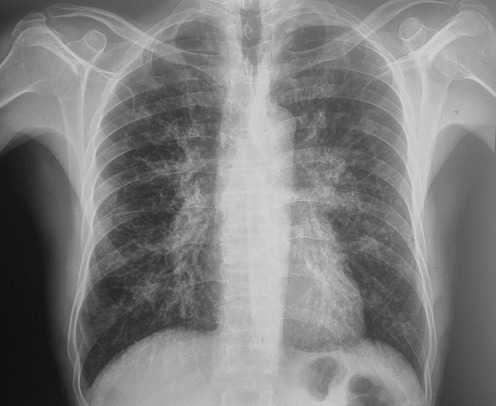
Radiographie standard du thorax

**Figure 2:**
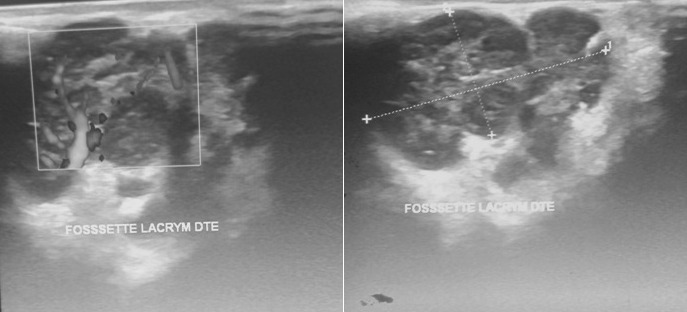
Echographie oculaire

**Figure 3:**
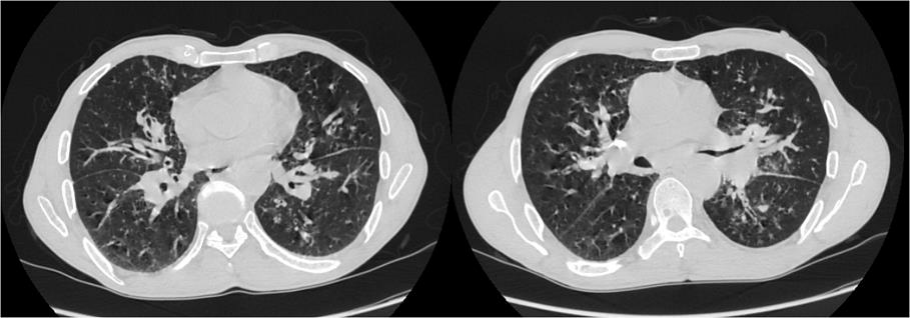
Scanner thoracique montrant un manchonnage tissulaire péri-bronchique bilateral

**Figure 4:**
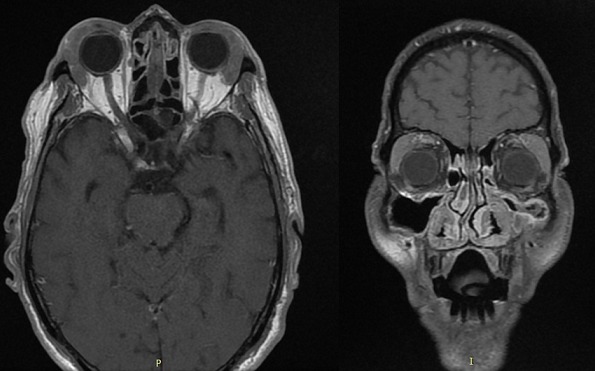
IRM orbitaire montrant une infiltration diffuse des deux glandes lacrymales

**Figure 5:**
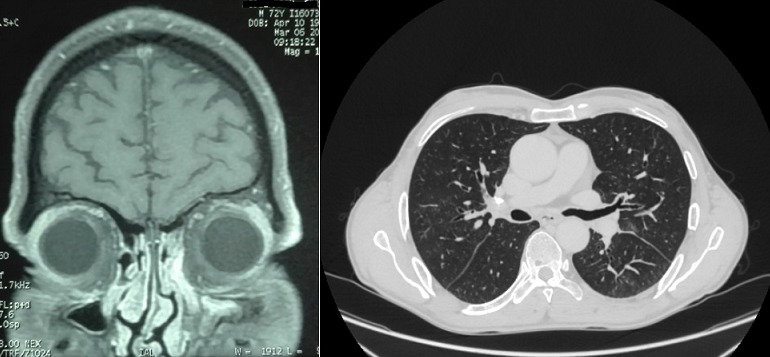
TDM thoracique et IRM orbitaire post-thérapeutique

Mr B.A âgé de 72 ans se présente avec un œdème palpébral, une obstruction nasale associés à une toux sèche et des crachats hémoptoiques évoluant depuis 6 mois. La radiographie thoracique avait révélé une atteinte micronodulaire, réticulaire pulmonaire bilatérale avec gros hiles (Figure 1). L'échographie oculaire a montré une glande lacrymale hypertrophiée d'échostructure hétérogène et richement vascularisée entrainant une discrète déformation du globe oculaire (Figure 2). Un scanner thoracique a été pratiqué et a montré un manchonnage tissulaire péri-bronchiquebilatéral associé à des micronodules de topographie lymphatique ainsi que des ganglions médiastinaux latéro-trachéaux droits et sous carinaires (Figure 3). L'IRM orbitaire a conclu à une tuméfaction et une infiltration diffuse des deux glandes lacrymales (Figure 4). Une fibroscopie bronchique avec biopsie a été pratiquée, l'examen anatomopathologique a montré une infiltration lymphomateuse à petites cellules B cadrant avec un lymphome type MALT CD20+ CD5- CK- bcl6- CD10-. Le bilan d'extension a été complété par une fibroscopie gastrique avec biopsie ayant objectivé une gastrite fundique chronique très active riche en Helicobacter Pylori. L'examen ORL et le scanner abdomino-pelvien étaient sans anomalies. Au bilan préthérapeutique les sérologies virales C, B et HIV étaient négatives.Le patient a été traité par 9 cycles Rituximab-Chlorambucil avec bonne tolérance. Il a également eu une quadrithérapie anti-Helicobacter pylori pour la gastrite fundique HP (+) avec contrôle son éradication. A la fin de la chimiothérapie, l'IRM orbitaire a montré une disparition quasi-complète de la tuméfaction et de l'infiltration diffuse des deux glandes lacrymales. La TDM thoraco-abdomino-pelvienne était normale (Figure 5). Une surveillance a été préconisée. Cinq mois après la fin du traitement le patient est toujours en rémission complète.

## Discussion

L'intérêt de cette observation est d'illustrer deux localisations rares et synchrones d'un lymphome de type MALT. En effet une présentation multifocale métachrone semble plus fréquente pour les lymphomes MALT extradigestif [3]. D'après la classification révisée de l'OMS le lymphome du MALT pulmonaire est un Lymphome de la zone marginale extra-ganglionnaire du tissu lymphoïde associé aux muqueuses [mucosa-associatedlymphoid tissue (MALT)] [4]. Le tabac ne semble pas être un facteur favorisant mais l'hypothèse d'une stimulation antigénique chronique (agent infectieux ou auto-Ac) est actuellement évoquée [1]. La coexistence d'un LNH de type MALT et d'un LNH de haut grade est rapporté sur certains terrains immuno-déprimés (traitements par immunosuppresseurs, VIH) ou présentant une maladie dysimmunitaire. Le lymphome MALT représente près de 85% des lymphomes primitifs pulmonaires. Il s'associe à des localisations extra-thoraciques dans 25 à 45 % des cas. Il n'y a pas de prédominance liée au sexe et touche généralement les sujets âgés entre 45 et 70 ans. Les localisations extra-pulmonaires les plus fréquemment décrites sont gastro-intestinales, et oculaires [1]. Les signes révélateurs du lymphome de MALT pulmonaire sont souvent discrets et peu spécifiques ce qui explique les difficultés du diagnostic. Il peut s'agir le plus souvent d'une toux, d'une dyspnée, de douleurs thoraciques ou d'une hémoptysie. Dans notre pays, peu de cas ont été rapportés [5]. La rareté du diagnostic souligne l'intérêt de cette observation dans notre pays où le diagnostic différentiel avec les cancers solides broncho-pulmonaires voire la tuberculose est loin d'être évident. La radiographie standard permet d'orienter le diagnostic en montrant souvent des opacités alvéolaires plus ou moins denses, localisées, à contours nets ou flous. La tomodensitométrie thoracique a une meilleure sensibilité que la radiographie standard. Contrairement au cancer broncho-pulmonaire, le scanner montre généralement des opacités alvéolaires multifocales comprenant un alvéolo-bronchogramme aérique. La localisation dans le lobe moyen est la plus fréquente. Les autres formes, moins fréquentes et moins caractéristiques, comportent: la présentation« pseudo-tumorale », avec une masse de taille comprise entre 2 et 25 cm, bien délimitée; et la forme «infiltrante», avec des opacités en verre dépoli diffuses, correspondant à un stade précoce de la maladie [6, 7]. L'apport de la TEP-FDG (tomographie par émission de positrons au 18-fluorodeoxyglucose) dans le bilan d'extension semble intéressant mais n'est pas encore évalué avec exhaustivité [8].

L'endoscopie bronchique est un examen fondamental dans la démarche diagnostique. Celle-ci a permis d'avoir le diagnostic de lymphome du MALT pulmonaire chez notre patient en montrant des lésions endobronchiques et en permettant les biopsies endoscopiques. L'examen histologique et immunohistochimique confirme le diagnostic en montrant le sous-type tumoralB de la prolifération lymphoïde intra-alvéolaire, avec la positivité des marqueurs pan-B CD20, CD79, et la négativité des marqueurs CD5 et CD10 [4]. Le bilan d'extension doit comporter un scanner cervico-thoraco-abdomino-pelvien, un examen ORL et ophtalmologique et une FOGD. Une biopsie ostéomédullaire peut être indiquée dans certains cas. Chez notre patient l'échographie oculaire et l'IRM orbitaire demandés devant la coexistence d'un œdème palpébral ont montré une glande lacrymale hypertrophiée et infiltrée confirmant la localisation lymphomateuse. Le reste du bilan d'extension était sans anomalies. Cependant la biopsie osteomédullaire n'a pas été pratiquée. Les lymphomes des annexes de l’œil ne représentent que 8% des lymphomesextra-ganglionnaires. Les tissus lymphoïdes composés de cellules B et T ne sont pas présents au niveau de l'orbite, hormis dans la conjonctive et la glande lacrymale [9]. Cette localisation lacrymale intéresse l'ensemble de la glande orbitaire et palpébrale. Son développement est lent, progressif et se fait sur un mode peu inflammatoire pendant des années. L'imagerie montre comme chez notre patient une tumeur intéressant l'ensemble de la glande, sans ostéolyse ni envahissement du globe oculaire et prenant la forme d'une virgule sur les coupes axiales. D'autres atteintes des annexes orbitaires sont possibles et notamment du muscle oblique inférieur et responsable d'une orbitopathie inflammatoire chronique. Une atteinte au niveau de l'orbite antérieure avec une extension épisclérale peut aussi se voir [9]. Les lymphomes extra-ganglionnaire de type MALT ont souvent un bon pronostic avec une survie supérieure à 5 ans dans plus de 80% des cas. En dehors des lymphomes de MALT digestifs, il n'existe pas encore stratégie thérapeutique standard consensuelle. Les types de prise en charge proposés varient entre la simple surveillance, la chirurgie, la mono- ou polychimiothérapie, l'immunothérapie ou la radiothérapie. L'emploi d'une chimiothérapie exclusive est admis en cas d'atteinte bilatérale ou extra-pulmonaire, de rechute ou de progression. Les recommandations actuelles sont plutôt en faveur d'une monothérapie par chloraminophène. La place du Rituximab n'est actuellement pas bien établie. En effet, le seul essai thérapeutique prospectif randomisé incluant les patients non répandants ou non éligibles à un traitement local et comparant : chloraminophène à l'association Chloraminophène/Rituximab a conclu à un gain significatif en survie sans événements à 5 ans de 18% mais sans impact sur la survie globale [10]. Notre patient a reçu 6 cycles de Rituximab associé au chloraminophène et depuis il est en rémission complète.

## Conclusion

Le diagnostic de lymphomes du MALT pulmonaires ne doit pas être méconnu. A travers notre observation, nous avons souligné l'apport de la fibroscopie bronchique qui a permis de faire les prélèvements biopsiques et apporter un diagnostic. Mais le manque de spécificité des signes cliniques et para-cliniques doit inciter à éliminer une tuberculose pulmonaire compte tenu de notre contexte d'endémie mais aussi un cancer broncho-pulmonaire. La recherche d'autres localisations extrapulmonaires, quoique rares, doit faire partie du bilan initial.

## Conflits d’intérêts

Les auteurs ne déclarent aucun conflit d'intérêts.
